# Screening of anti-heart failure active compounds from fangjihuangqi decoction in verapamil-induced zebrafish model by anti-heart failure index approach

**DOI:** 10.3389/fphar.2022.999950

**Published:** 2022-10-07

**Authors:** Jun Li, Yue Zhu, Xiaoping Zhao, Lu Zhao, Yi Wang, Zhenzhong Yang

**Affiliations:** ^1^ Pharmaceutical Informatics Institute, College of Pharmaceutical Sciences, Zhejiang University, Hangzhou, China; ^2^ School of Basic Medical Sciences, Zhejiang Chinese Medical University, Hangzhou, China; ^3^ Innovation Institute for Artificial Intelligence in Medicine of Zhejiang University, Hangzhou, China; ^4^ Jinhua Institute of Zhejiang University, Jinhua, China; ^5^ Innovation Center in Zhejiang University, State Key Laboratory of Component-Based Chinese Medicine, Hangzhou, China

**Keywords:** fangjihuangqi decoction, heart failure, zebrafish, oxidative stress, inflammatory response, apoptosis

## Abstract

Heart failure is the end stage of various cardiovascular diseases. Fangjihuangqi Decoction (FJHQD) is a famous traditional Chinese medicine (TCM) formula, which is clinically effective in the treatment of chronic heart failure. However, the anti-heart failure ingredients of FJHQD have not been clarified, and the related mechanisms of action are rarely studied. In the present study, through quantification analysis of heart rate and ventricular area changes, a heart failure model and cardiac function evaluation system in cardiomyocytes-labelled *Tg (cmlc2: eGFP)* transgenic zebrafish larvae were constructed, and the anti-heart failure index (AHFI) that can comprehensively evaluate the cardiac function of zebrafish was proposed. Based on this model, FJHQD, its mainly botanical drugs, components and ingredients were evaluated for the anti-heart failure effects. The results showed that FJHQD and its botanical drugs exhibited potent anti-heart failure activity. Furthermore, total alkaloids from *Stephania tetrandra* S. Moore, total flavonoids from *Astragalus mongholicus* Bunge and total flavonoids from *Glycyrrhiza uralensis* Fisch. ex DC. were identified to be the main components exerting the anti-heart failure activity of FJHQD. Then, we screened the main ingredients of these components, and glycyrrhizic acid, licochalcone A and calycosin were found to exhibit excellent cardioprotective effects. Finally, we found that FJHQD, glycyrrhizic acid, licochalcone A and calycosin may improve cardiac function in zebrafish by regulating oxidative stress, inflammatory response and apoptosis-related pathways. Taken together, our findings offer biological evidences toward the anti-heart failure effect of FJHQD, and provide guidance for the clinical application of FJHQD.

## Introduction

Cardiovascular disease (CVD) is one of the leading causes of death all over the world, responsible for about 9.6 million deaths per year among men and 8.9 million women, accounting for about one-third of all deaths from diseases, while China has the highest number of CVD deaths (Roth et al., 2020). Heart failure is a clinical syndrome characterized by fatigue and dyspnea caused by left (or systemic) ventricular dysfunction, often with signs of congestion (Rossignol et al., 2019; McDonagh et al., 2021), which is the terminal stage of various cardiovascular diseases. Conventional pharmacological treatment for heart failure includes angiotensin converting enzyme inhibitors (ACEIs), β-blockers, diuretics, corticosteroid receptor antagonists, sodium-glucose cotransporter two inhibitors and so on (Gupta et al., 2014). Despite numerous drugs available, the morbidity and mortality of heart failure remain high (Rossignol et al., 2019). Therefore, how to effectively mitigate disease progression and reduce the morbidity and mortality of heart failure has become a global public health problem.

Traditional Chinese medicine (TCM) provides rich resource for bioactive components discovery and novel pharmacological therapy development (Tu, 2011; Yang et al., 2018a; Yang et al., 2018b). The development of medicine for treating heart failure from natural products has aroused great enthusiasm. FJHQD is a famous TCM formula mainly composed of *Stephania tetrandra* S. Moore [Menispermaceae; Stephaniae Tetrandrae Radix] (STR), *Astragalus mongholicus* Bunge [Fabaceae; Astragali Radix] (AR), *Atractylodes macrocephala* Koidz [Asteraceae; Atractylodis Macrocephalae Rhizoma] (AMR), and *Glycyrrhiza uralensis* Fisch. ex DC [Fabaceae; Glycyrrhizae Radix et Rhizoma] (GRR), which is prescribed to treat chronic heart failure, chronic glomerulonephritis, cardiogenic edema, and rheumatoid arthritis embolism (Zhang et al., 2019; Hwa et al., 2021). However, due to the complicated composition in naturally derived materials, how FJHQD and the bioactive ingredients prevent heart failure is still largely unclear.

Zebrafish is a model organism with powerful advantages making it particularly attractive for biomedical researchers. As an experimental vertebrate, zebrafish is more relevant to human than the nematode *Caenorhabditis elegans* and the fruit fly *Drosophila melanogaster*, but of relatively lower cost, smaller size and rapider development compared with the murine model *Mus musculus* (Giacomotto and Segalat, 2010). The heart initiates heartbeat from 24 h postfertilization (hpf), and is fully formed by 48 hpf. Additionally, the embryos and larvae are nearly transparent, making it possible to observe the internal structures, such as heart, in real time without invasive instrumentation. Zebrafish has been used as a model organism for cardiovascular toxicity or activity screening (Bournele and Beis, 2016). Several chemical-induced heart failure models in zebrafish have been reported (Shi et al., 2018; Zhu et al., 2018). In this study, we aim to establish the verapamil-induced heart failure model in zebrafish larvae and use it for the evaluation of the anti-heart failure activity of FJHQD and its active ingredients screening. The therapeutic mechanisms of FJHQD and its active ingredients were also explored.

## Materials and methods

### Chemicals and reagents

AR was collected from the Inner Mongolia Autonomous Region, China. AMR and STR were collected from Zhejiang Province, China. GRR was collected from the Xinjiang Uygur Autonomous Region, China. *Zingiber officinale* Roscoe [Zingiberaceae; Zingiberis Rhizoma Recens] (ZRR) was collected from Yunnan Province, China. *Ziziphus jujuba* Mill [Rhamnaceae; Jujubae Fructus] (JF) was collected from Hebei Province, China. Voucher specimens were deposited in Pharmaceutical Informatics Institute, Zhejiang University, and morphologically identified by Dr. Zhenzhong Yang.

Licochalcone A, fangchinoline, tetrandrine, liquiritin apioside, isoliquiritin apioside, calycosin, formononetin, ononin, glycyrrhetinic acid and isoliquiritin were obtained from Shanghai Yuanye Biotechnology Co., Ltd. (Shanghai, China). Calycosin-7-O-glucoside, liquiritigenin, liquiritin and isoliquiritigenin were purchased from Shanghai Ronghe Pharmaceutical Technology Development Co., Ltd. (Shanghai, China). Licochalcone B and glycyrrhizic acid were purchased from Chengdu Must Bio-Technology Co., Ltd (Chengdu, China). The purity of all reference standards was more than 97%. Verapamil and DCFH-DA were obtained from Dalian Meilun Biotechnology Co., Ltd. (Dalian, China). N-Phenylthiourea (PTU) was purchased from Sigma-Aldrich (St. Louis, MO, United States). Dimethyl sulfoxide (DMSO) was obtained from Sinopharm Chemical Reagent Co., Ltd. (Shanghai, China).

### Sample preparation

FJHQD extract preparation: The preparation of FJHQD extract has been reported in our previous study (Yang et al., 2022). STR, AR, AMR, GRR, ZRR and JF were mixed at a ratio of 60:75:45:30:45:36, and then extracted twice with water (1:8, m/V) for 1 h under reflux. The extract was combined and concentrated under 70°C and the reduced pressure to dry, and then re-suspended in DMSO at a concentration of 500 mg/ml.

Extracts preparation of botanical drug: The preparation process is similar with FJHQD. The individual botanical drug was extracted twice with water (1:8, m/V) for 1 h under reflux. The extract was combined and concentrated under 70°C and the reduced pressure to dry, and then re-suspended in DMSO at a concentration of 500 mg/ml.

Main components preparation: Herbal extracts were loaded onto the macroporous resin, eluted with water and ethanol solutions of different concentrations. The eluates containing the corresponding components were collected, combined, and concentrated to dry. The components were stored for the further experiments.

### Zebrafish husbandry

Wildtype AB strain and *Tg(cmlc2: eGFP) (* Kwan et al., 2007) transgenic zebrafish were all obtained from the Laboratory Animal Center of Zhejiang University. Zebrafish were maintained following standard protocols. E3 medium (0.29 g/L NaCl, 0.013 g/L KCl, 0.048 g/L CaCl_2_.2H_2_O, 0.082 g/L MgCl_2_.6H_2_O, pH 7.2) was used as the zebrafish medium. Embryos were obtained through natural spawning.

### Zebrafish embryo collection and sample treatment

Adult zebrafish were used to generate zebrafish embryos. Fertilized embryos were selected by microscopic examination and then raised in embryo medium containing PTU (0.2 mM) at 28°C for 2 days. Zebrafish larvae of 3 days post fertilization (dpf) were collected into a 12-well microplate (12–15 fish per well), and were treated with FJHQD for another 4 h. DMSO (0.2% *v/v* final concentration) was used as negative control. At the 76 hpf stage, the larvae were washed three times with fish water, and treated with verapamil (200 µM) for 30 min. After verapamil treatment, the larvae were anesthetized and transferred into 96-well microplate (one fish per well), and images were acquired under Leica DMI 3000 B inversed microscope system (Leica Microsystems Inc., United States).

### Image analysis-based quantitative phenotyping of cardiac functions in zebrafish.

Heart rate (HR), fractional area change (FAC), stroke volume (SV), fractional shortening (FS), ejection fraction (EF) and cardiac output (CO) were selected as indicators of cardiac function (Ho et al., 2007; Shin et al., 2010). In order to accurately assess the cardiac function in zebrafish, we calculated the HR and selected frames at the end systole (ES) phase and end diastole (ED) phase from the sequence images. With the endocardium bounded, the heart is defined, and its area, as well as the long and short axis, was measured. The indicators of cardiac function were calculated *via* the following formulae:

$$FAC=\frac{EDA-ESA}{EDA}\times 100\%$$

*EDA*: the heart area of ED; *ESA*: the heart area of ES.

$$FS=\frac{D_{d}-D_{s}}{D_{d}}\times 100\%$$

*D*_*d*_: short axis at ED; *D*_*s*_: short axis at ES.

$$The\;Ventricular\;volume=\frac{4}{3}\times \pi \times \frac{1}{2}a\times \frac{1}{4}b^{2}$$

*a* and *b* are the half-long and half-short axis of heart, respectively.

SV=EDV−ESV

*EDV*: the ventricular volume of ED; *ESV*: the ventricular volume of ES.

$$EF=\frac{SV}{EDV}\times 100\%$$

*EDV*: the ventricular volume of ED.

CO=SV×HR

### Reactive oxygen species measurement

Zebrafish larvae were incubated with 10 μM of DCFH-DA solution for 30 min at 28.5°C in the dark. After that, larvae were washed with E3 medium and imaged with Leica DMI 3000 B. The fluorescence intensity of individual larvae was quantified using the ImageJ program (v1.8.0).

### HPLC analysis

According to the method of our previous research, we performed HPLC analysis of the components of FJHQD (Yang et al., 2022). In brief, chromatographic separations were carried out at 30°C on a CORTECS C_18_ column (4.6 mm × 100 mm, 2.7 mm) with acidified water (0.1% formic acid) and acetonitrile as mobile phases A and B, respectively. The solvent gradient adopted was as follows: 0 min, 2%B; 4 min, 5%B; 10 min, 12%B; 15 min, 15%B; 20 min, 20% B; 25 min, 25% B; 30 min, 33%B; 35 min, 45%B; 45 min, 70% B; 50 min, 100%B. The flow rate was 0.7 ml/min, the detection wavelength was 254 nm, and the injection volume was 5 μL.

### mRNA extraction and qPCR

Zebrafish larvae were transferred to 6-well plates with 30 larvae per well, and total RNA was extracted using the RNA Rapid Extraction Kit, followed by reverse transcription into single-stranded cDNA using the HiFiScript cDNA Synthesis Kit (ES Science). cDNA was stored at -20°C. Fluorescent quantitative PCR was performed using 2 × SYBR Green qPCR Mater Mix (Bimake). Primer sequences are shown in Table 1.

**TABLE 1:** The Sequences of all primers used in the study.

Gene	Forward primer	Reverse primer
*ef1a*	AGA​AGG​CTG​CCA​AGA​CCA​AG	AGA​GGT​TGG​GAA​GAA​CAC​GC
*tnfa*	GCT​TAT​GAG​CCA​TGC​AGT​GA	TGC​CCA​GTC​TGT​CTC​CTT​CT
*il1b*	GTC​ACA​CTG​AGA​GCC​GGA​AG	GCA​GGC​CAG​GTA​CAG​GTT​AC
*il6*	ACG​ACA​TCA​AAC​ACA​GCA​CC	TCG​ATC​ATC​ACG​CTG​GAG​AA
*caspase 1*	GTG​GTC​ACC​GAA​TGC​CAG​TA	TCG​CAG​CAA​GGT​TTT​CCT​CT
*caspase 3*	CCGCTGCCCATCACTA	ATCCTTTCACGACCATCT
*nppa*	GAT​GTA​CAA​GCG​CAC​ACG​TT	TCT​GAT​GCC​TCT​TCT​GTT​GC

### Anti-heart failure index calculation

The data normalization method is given below using HR as an example.

$$B_{HR,i}=\frac{{HR}_{i}}{{HR}_{ctrl}}$$

*HR_i_*: heart rate of ingredient *i* treated group; *HR_ctrl_*: heart rate of the control group. The data normalization method of the rest cardiac function indicators is calculated in a similar way to HR.

The formula for calculating the recovery score of cardiac function (RCF) is given below using HR as an example.

$${RCF}_{HR,i}=\frac{B_{HR,i}-B_{HR,m}}{1-B_{HR,m}}\times 100\%$$

${RCF}_{HR,i}$ : recovery score of ingredient *i* in HR; $B_{HR,i}$ : normalized HR of ingredient *i* treated group; $B_{HR,m}$ : normalized HR of verapamil-induced group. The recovery scores of the rest cardiac function indicators are calculated in a similar way to heart rate.

Considering the correlation of the cardiac function indicators, four relatively independent recovery scores of HR, EF, CO and FAC, were selected for the calculation of the anti-heart failure index (AHFI), which was calculated as follows:

$${AHFI}_{i}={RCF}_{HR,i}+{RCF}_{EF,i}+{RCF}_{CO,i}+{RCF}_{FAC,i}$$

*AHFI*_*i*_: anti-heart failure index of ingredient *i*; ${RCF}_{HR,i}$ : heart rate recovery score of ingredient *i*; ${RCF}_{EF,i}$ : ejection fraction recovery score of ingredient *i*; ${RCF}_{CO,i}$ : cardiac output recovery score of ingredient *i*; ${RCF}_{FAC,i}$ : fractional area change recovery score of ingredient *i*.

### Statistics

All data are presented as the mean ± standard deviation (SD). Differences between two groups were analyzed using the two-tailed Student’s t-test. Multiple group comparison was performed by one-way ANOVA. The *p* value < 0.05 was considered statistically significant.

## Results

### Verapamil-induced heart failure model in zebrafish

**FIGURE 1:**
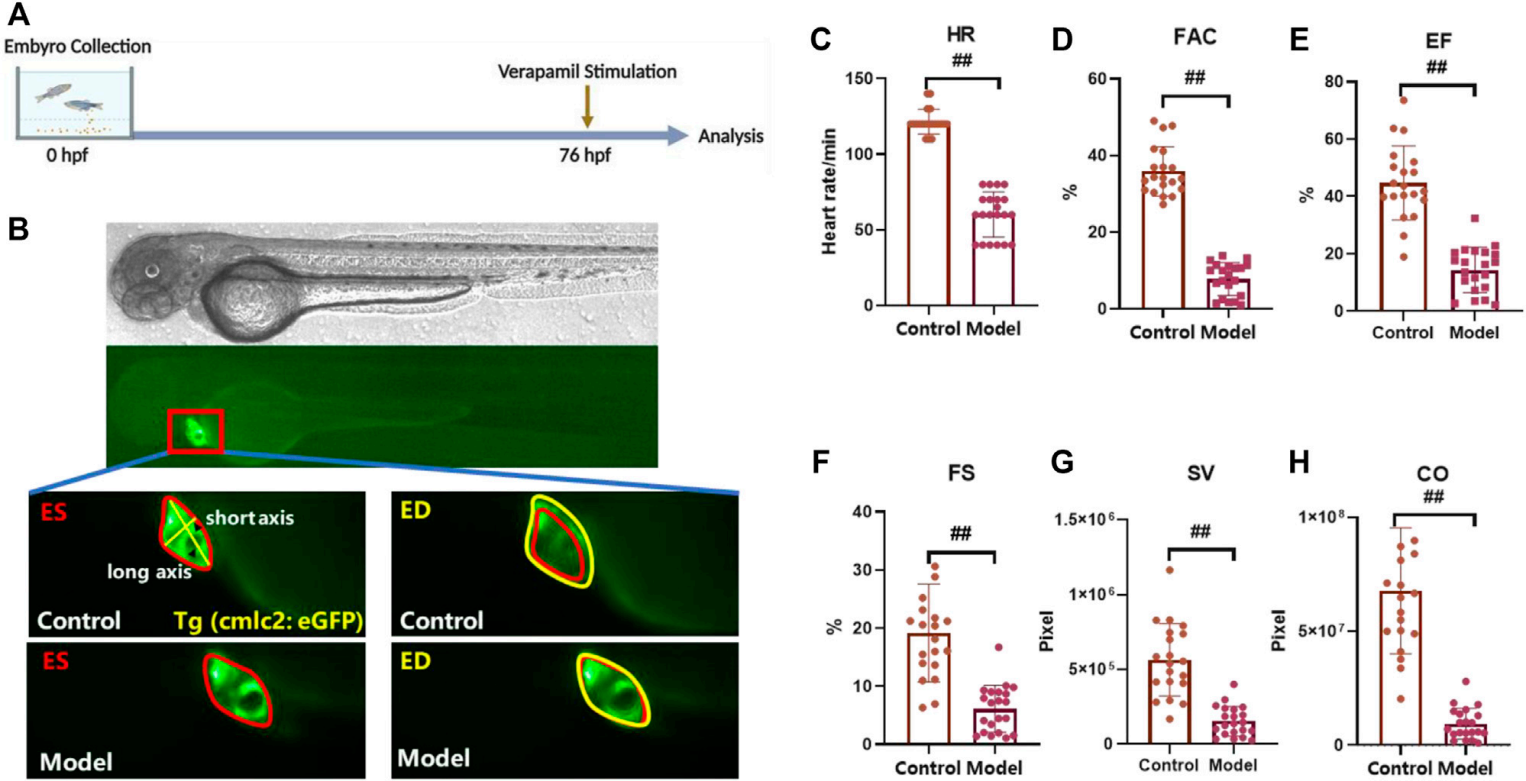
Verapamil-induced heart failure model in zebrafish **(A)** Experimental scheme for heart failure model in zebrafish. **(B)** Representative images of vehicle-treated and verapamil-treated zebrafish heart. The heart of ES was shown in red line, and the heart of ED was shown in yellow line; ES: end systole, ED: end diastole **(C–H)** Quantification of cardiac function in *Tg(cmlc2: eGFP)* zebrafish larvae with verapamil. HR: heart rate, FAC: fractional area change, SV: stroke volume, FS: fractional shortening, EF: ejection fraction, CO: cardiac output. N ≥ 20. ^#^ *p* < 0.05 vs*.* Control, ^##^ *p* < 0.01 vs*.* Control.

As previously reported, verapamil leads to decreased cardiac function and venous stasis in zebrafish larvae, which is largely consistent with the symptoms of heart failure in humans. Therefore, we chose verapamil to build a heart failure model with cardiomyocytes-labelled *Tg(cmlc2: eGFP)* transgenic zebrafish (Zhu et al., 2018; Li et al., 2021). Verapamil at the concentration of 200 µM was adopted to treat zebrafish larvae at 76 hpf for 30 min (Figure 1A). Then, under the microscope, it can be observed that the zebrafish heart rate generally slowed down (Figure 1C) and the contractile ability of the heart was obviously weakened (Figure 1B). After analyzing the sequence images of zebrafish heart, the parameters indicating the contractile function, i.e., FAC, EF, FS, SV and CO, were significantly reduced in the verapamil-treated group (Figures 1B–H). In this model, the heart rate and cardiac output reduced, which depicted the zebrafish heart failure model was developed (Zhu et al., 2018). Thus, we continued to examine the efficacy of FJHQD, its botanical drugs, components and ingredients on this model.

### FJHQD alleviates cardiac function in verapamil-induced heart failure model

**FIGURE 2:**
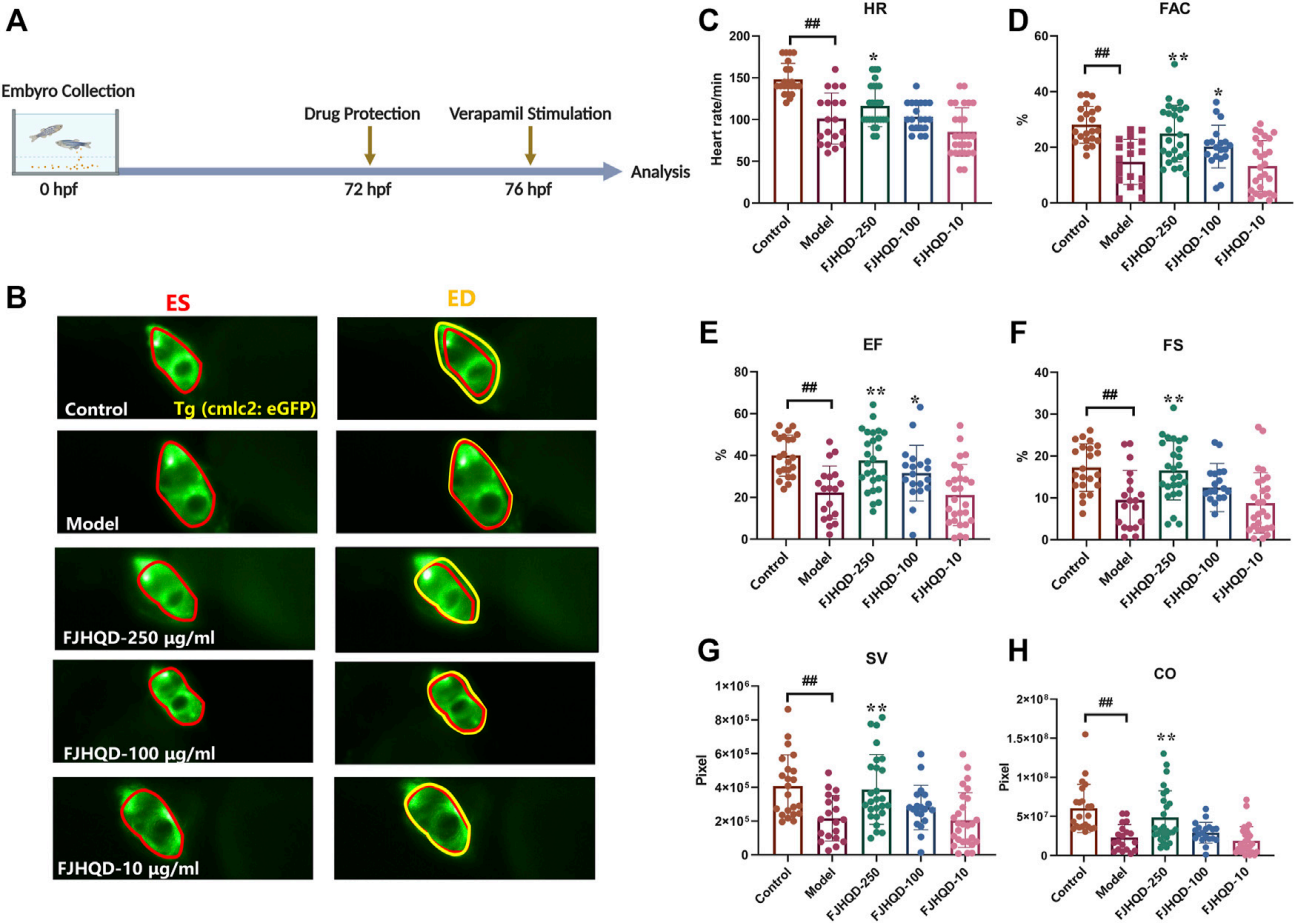
FJHQD alleviates cardiac function in verapamil-induced heart failure model **(A)** Experimental scheme for heart failure model in zebrafish. **(B)** Representative images of vehicle-treated and verapamil-treated zebrafish heart. The heart of ES was shown in red line, the heart of ED was shown in yellow line **(C–H)** Quantification of cardiac function in *Tg(cmlc2: eGFP)* zebrafish larvae treated with FJHQD. n ≥ 19. ^#^ *p* < 0.05 vs*.* Control, ^##^ *p* < 0.01 vs*.* Control, * *p* < 0.05 vs*.* Model, ** *p* < 0.01 vs*.* Model.

Toxicity analysis suggested that within a range of 10 μg/ml to 250 μg/ml, FJHQD incubation from 72 to 76 hpf showed no significant pericardial edema, bradycardia or death on the survival or general development of zebrafish larvae, so the maximum concentration for subsequent studies was set to be 250 μg/ml. Next, we added the FJHQD treatment before verapamil stimulation, and evaluated its effects on cardiac function (Figures 2A,B). Through quantification analysis of zebrafish ventricular area by fluorescent sequence imaging, a significant restoration of cardiac function was observed in the larvae protected by FJHQD in a dose-dependent manner, with 250 μg/ml concentration providing the best rescue effects (Figures 2C–H).

### The therapeutic effects of botanical drugs on verapamil-induced heart failure model in zebrafish

**FIGURE 3:**
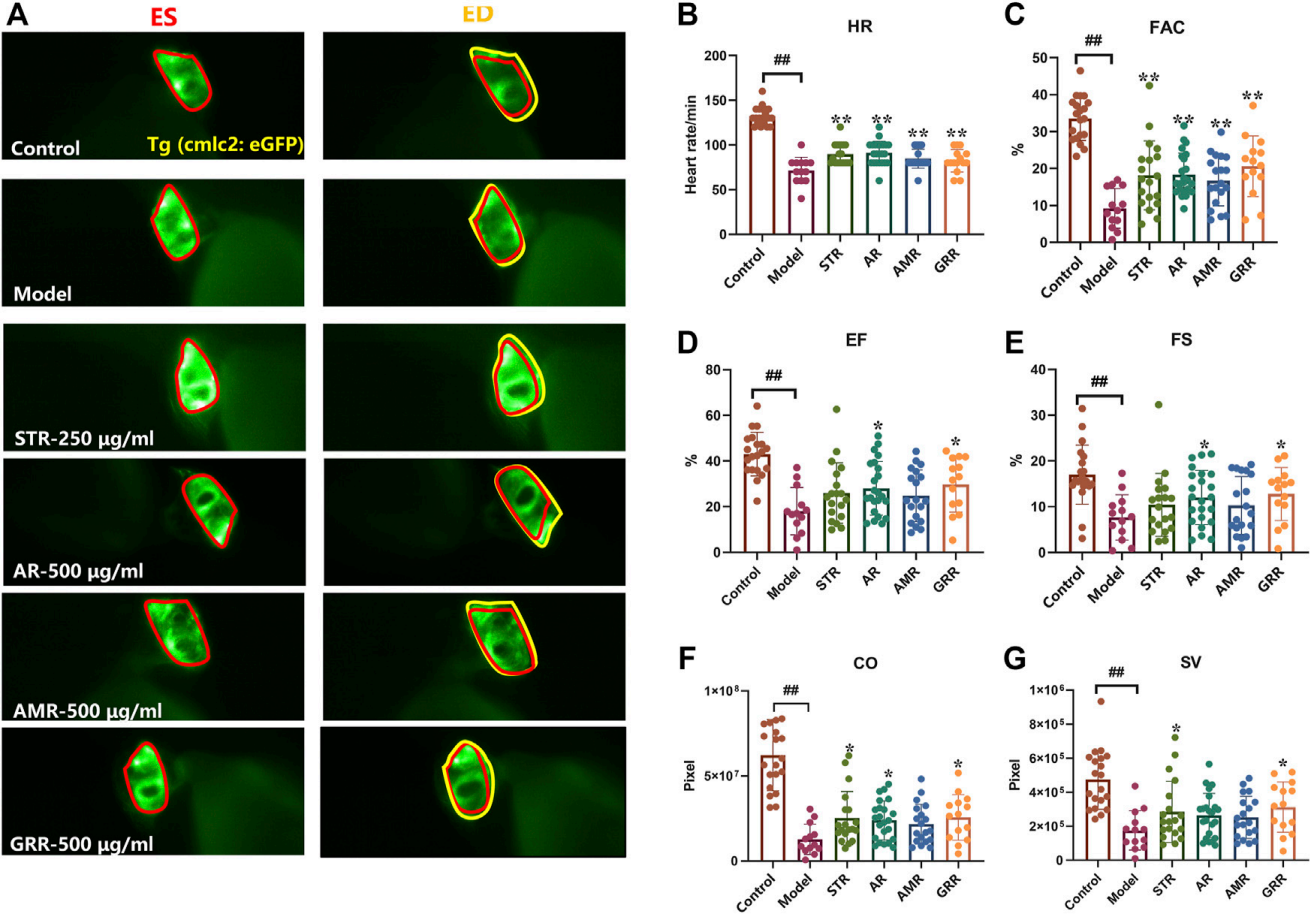
The therapeutic effects of botanical drugs on verapamil-induced heart failure model in zebrafish. Representative images **(A)** and quantification **(B–G)** of cardiac function in *Tg (cmlc2: eGFP)* zebrafish larvae treated with botanical drugs. N ≥ 15. ^#^ *p* < 0.05 vs*.* Control, ^##^ *p* < 0.01 vs*.* Control, * *p* < 0.05 vs*.* Model, ** *p* < 0.01 vs*.* Model.

FJHQD is a famous TCM formula mainly composed of STR, AR, AMR and GRR, which is clinically effective in the treatment of chronic heart failure. To investigate the botanical drugs in FJHQD that exert anti-heart failure activity, the extracts of individual botanical drug were prepared and evaluated for their anti-heart failure effects in the zebrafish heart failure model. Markedly, compared with model group, GRR treatment could improve all cardiac function indicators in zebrafish. Moreover, STR and AR also exhibited remarkable anti-heart failure activity in most cardiac function indicators (Figures 3B–G). These results suggested that STR, AR and GRR may be the botanical drugs that contribute the anti-heart failure activity of FJHQD.

**FIGURE 4:**
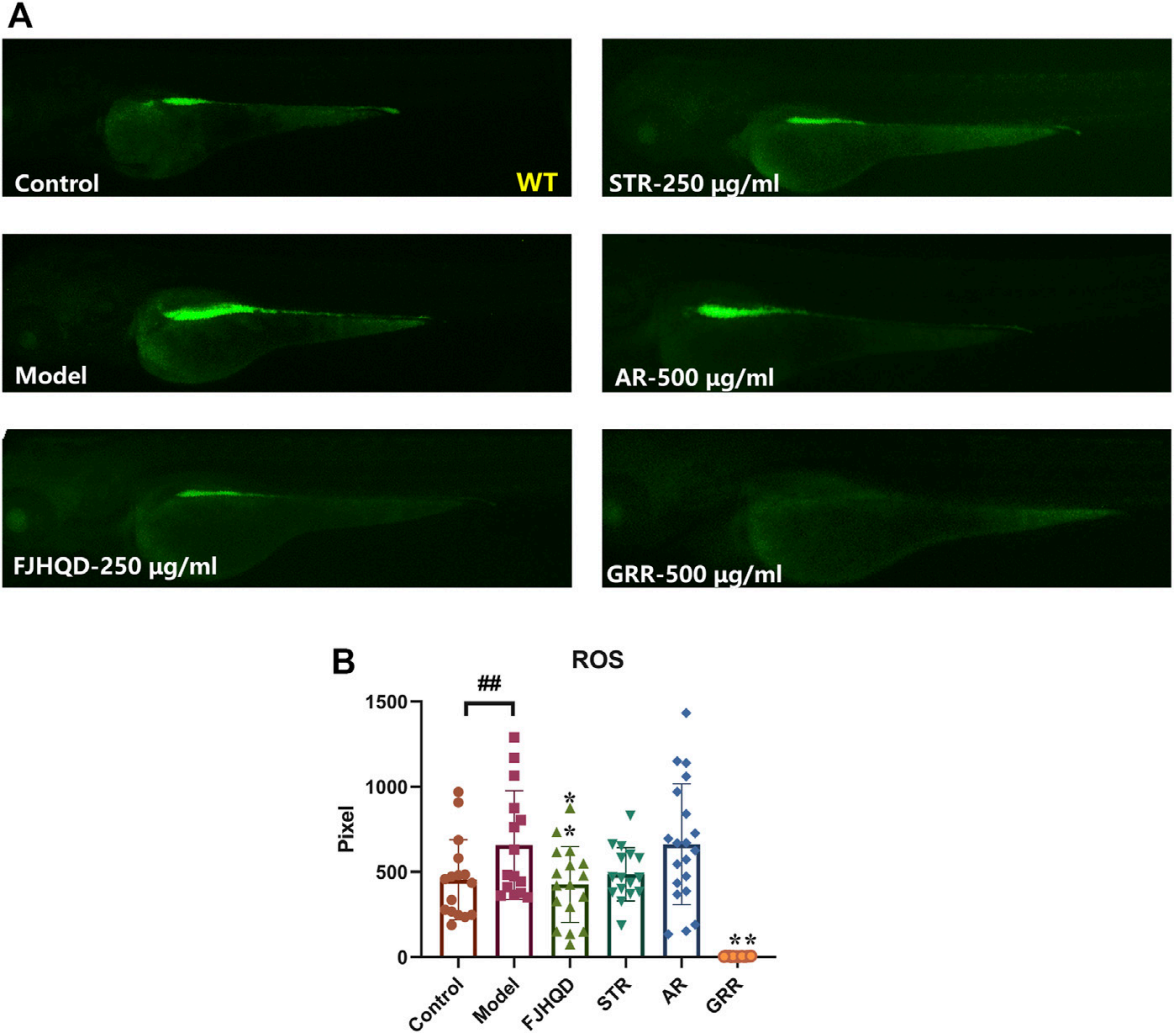
FJHQD, STR and GRR inhibit oxidative damages in verapamil-induced heart failure zebrafish. Representative images **(A)** and quantification **(B)** of the fluorescence signals of DCFH-DA probe in control or verapamil treated larvae with or without botanical drugs extracts. N ≥ 11. ^#^ *p* < 0.05 vs*.* Control, ^##^ *p* < 0.01 vs*.* Control, * *p* < 0.05 vs*.* Model, ** *p* < 0.01 vs*.* Model.

Many studies have demonstrated the accumulation of ROS in the cardiovascular system is a response to various stresses and heart failure (Giordano, 2005; D'Aiuto et al., 2010; Bondia-Pons et al., 2012; Liu et al., 2016; Ojha et al., 2016; Singh et al., 2016; van der Pol et al., 2019). In addition, antioxidants can ameliorate ROS-mediated cardiac abnormalities (Takimoto and Kass, 2007). In zebrafish treated with verapamil, endogenous ROS production was significantly higher than in the control group. After pre-treatment with FJHQD, STR or GRR, ROS was significantly reduced compared to the model group, but not with AR. This suggested that the anti-heart failure activity of FJHQD and GRR may be, at least partly, achieved by reducing ROS production (Figures 4A,B).

### The therapeutic effects of the main components of botanical drugs on verapamil-induced heart failure model in zebrafish.

**FIGURE 5:**
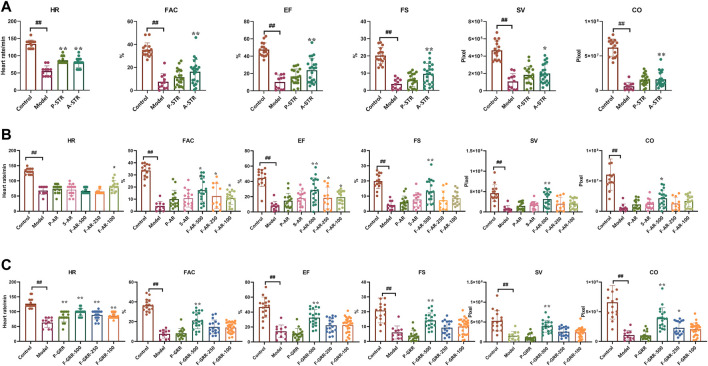
The therapeutic effects of the main components of botanical drugs on verapamil-induced heart failure model in zebrafish **(A–C)** Quantification of cardiac function in *Tg(cmlc2: eGFP)* zebrafish larvae treated with main components of STR, AR and GRR. N ≥ 10. ^#^ *p* < 0.05 vs*.* Control, ^##^ *p* < 0.01 vs*.* Control, * *p* < 0.05 vs*.* Model, ** *p* < 0.01 vs*.* Model.

In order to further search for components that exert anti-heart failure activity, the main components of extract of botanical drug were prepared, and evaluated for their anti-heart failure activity in the zebrafish model. The main components from STR include total polysaccharides of STR (P-STR) and total alkaloids of STR (A-STR). A-STR with the concentration of 100 μg/ml could significantly alleviate the decline in cardiac function caused by verapamil in zebrafish, while P-STR showed no obvious effect (Figure 5A). The main components from AR include total polysaccharides of AR (P-AR), total saponins of AR (S-AR), and total flavonoids of AR (F-AR). F-AR could dose-dependently alleviate most cardiac function indicators, including FAC, EF and SV, while S-AR and P-AR did not exhibit anti-heart failure activity (Figure 5B). The main components from GRR include total polysaccharides of GRR (P-GRR), total flavonoids of GRR (F-GRR), and saponins (mainly including glycyrrhizic acid and glycyrrhetinic acid). Interestingly, F-GRR showed dose-dependent therapeutic effects on all cardiac function indicators, while P-GRR didn’t exhibit anti-heart failure activity (Figure 5C). The anti-heart failure activities of glycyrrhetinic acid and glycyrrhizic acid were evaluated separately along with other ingredients.

### Characterization of chemical ingredients in the main active components of FJHQD

The chemical ingredients in A-STR, F-AR and F-GRR were analyzed using HPLC. The main chemical ingredients in A-STR were tetrandrine and fangchinoline (Supplementary Figure S1) (Tsutsumi et al., 2003; Sim et al., 2015; Xiao et al., 2018). Calycosin, calycosin-7-O-glucoside, ononin and formononetin were the main ingredients in F-AR (Supplementary Figure S2) (Su et al., 2021). The main chemical ingredients in F-GRR included liquiritigenin, liquiritin, isoliquiritigenin, isoliquiritin, liquiritin apioside, isoliquiritin apioside, licochalcone A and licochalcone B (Supplementary Figure S3) (Wang et al., 2012; Wang et al., 2020). The above results were consistent with our previous identification of the chemical ingredients in FJHQD (Yang et al., 2022), and these ingredients were selected for further study.

### The therapeutic effects of main ingredients in active components on verapamil-induced heart failure model in zebrafish.

**FIGURE 6:**
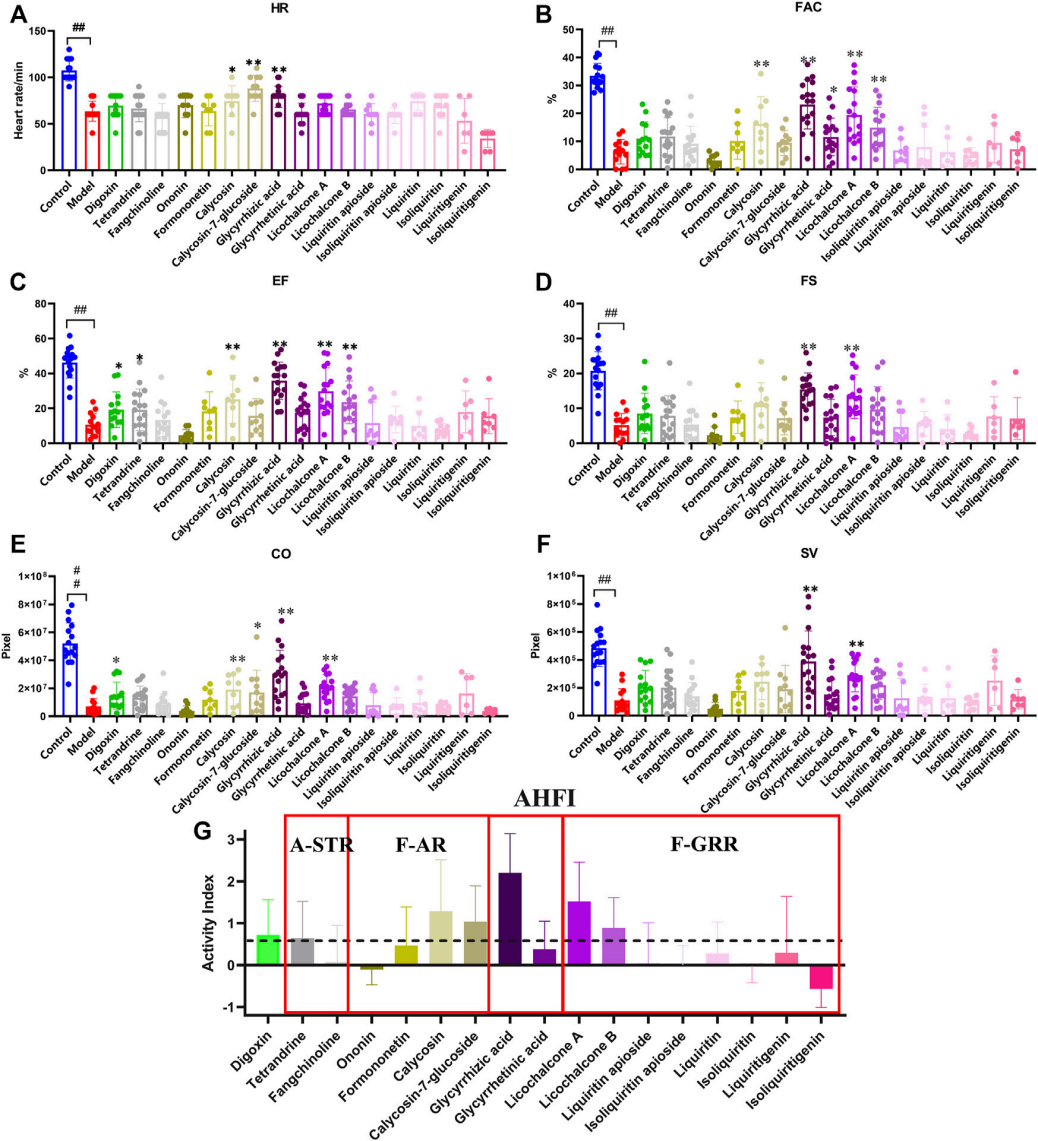
The therapeutic effects of main ingredients in active components on verapamil-induced heart failure model in zebrafish **(A–F)** Quantification of cardiac function in *Tg(cmlc2: eGFP)* zebrafish larvae treated with ingredients. **(G)** Quantification indicators of AHFI, the dotted line indicates an AHFI value of 0.6. N ≥ 6. ^#^ *p* < 0.05 vs*.* Control, ^##^ *p* < 0.01 vs*.* Control, * *p* < 0.05 vs*.* Model, ** *p* < 0.01 vs*.* Model.

We continued to screen for the active ingredients with anti-heart failure effects in the zebrafish heart failure model. The results showed that tetrandrine (100 μM), glycyrrhizic acid (100 μM), licochalcone A (1 μM), licochalcone B (50 μM) and calycosin (50 μM) could restore various cardiac function indicators to a certain extent (Figures 6A–F). However, some of the ingredients showed different alleviating effects in different cardiac function indicators, making it difficult to compare the anti-heart failure activity between them. AHFI was proposed herein to comprehensively evaluate the anti-heart failure activity of each ingredient, and its calculation was illustrated in Materials and Methods. The AHFI of the positive drug digoxin (10 μM) was about 0.7. Glycyrrhizic acid (100 μM), licochalcone A (1 μM) and calycosin (50 μM) showed excellent anti-heart failure activities with AHFI ≥1.0, while tetrandrine (100 μM), licochalcone B (50 μM) and calycosin-7-glycoside (50 μM) also had a certain degree of anti-heart failure activities (AHFI ≥0.6). These ingredients may be the main active compounds responsible for the anti-heart failure activity of FJHQD. Notably, among these ingredients, the anti-heart failure activities of licochalcone A and licochalcone B were firstly reported. In addition, the negative value of isoliquiritigenin (AHFI < -0.5) was also noteworthy. We comprehensively analyzed all six cardiac function indicators of isoliquiritigenin group, and found that five of them were close to those in model group, while the HR values were much lower. The HR reduced effect is one of the pharmacological effects of β-blockers, a currently used therapeutic drug for heart failure (Gupta et al., 2014). Further studies were needed to investigate the exact effect of isoliquiritigenin against heart failure.

### FJHQD and its active ingredients showed regulatory effects on oxidative stress, inflammatory response and apoptosis.

**FIGURE 7:**
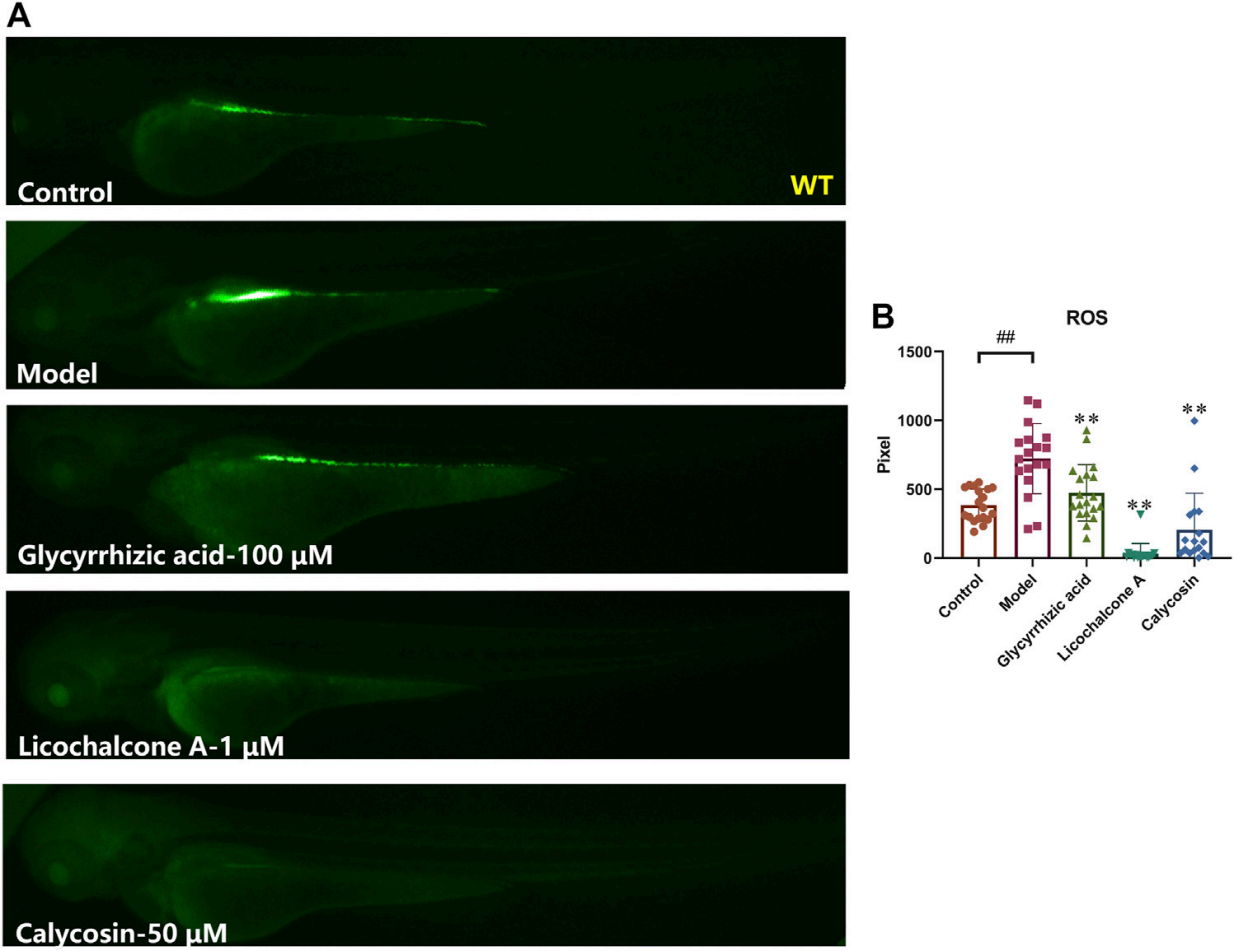
Glycyrrhizic acid, licochalcone A and calycosin inhibit oxidative damages in verapamil-induced heart failure zebrafish. Representative images **(A)** and quantification **(B)** of the fluorescence intensities of DCFH-DA probe in Control larvae or verapamil treated larvae with or without sample protection. N ≥ 15. ^#^ *p* < 0.05 vs*.* Control, ^##^ *p* < 0.01 vs*.* Control, * *p* < 0.05 vs*.* Model, ** *p* < 0.01 vs*.* Model.

In the heart, excess ROS can lead to maladaptive myocardial remodeling and progression of heart failure. ROS directly impair the electrophysiology and contractile mechanisms of cardiomyocytes by modifying core proteins of excitation-contraction coupling, including L-type calcium channels, sodium channels, potassium channels, and sodium-calcium exchangers (Takimoto and Kass, 2007; van der Pol et al., 2019). To further investigate whether the above-mentioned active ingredients against heart failure could reduce ROS content in zebrafish, the ROS fluorescent probe was photographed after co-incubation with verapamil for 30 min with zebrafish after 4 h of sample pre-treatment, and the fluorescent area was statistically analyzed. The results showed that glycyrrhizic acid (100 μM), licochalcone A (1 μM) and calycosin (50 μM) could significantly reduce the ROS elevation in zebrafish caused by verapamil. Among them, glycyrrhizic acid, licochalcone A and calycosin treatment group were even lower than the control group, showing their powerful antioxidant activities (Figure 7), which is consistent with previous reports (Haleagrahara et al., 2011; Liu et al., 2016; Ojha et al., 2016; Huang et al., 2019). The above results suggested that glycyrrhizic acid, licochalcone A and calycosin can scavenge endogenous ROS in zebrafish, or improve the anti-oxidant capacity of zebrafish.

**FIGURE 8:**
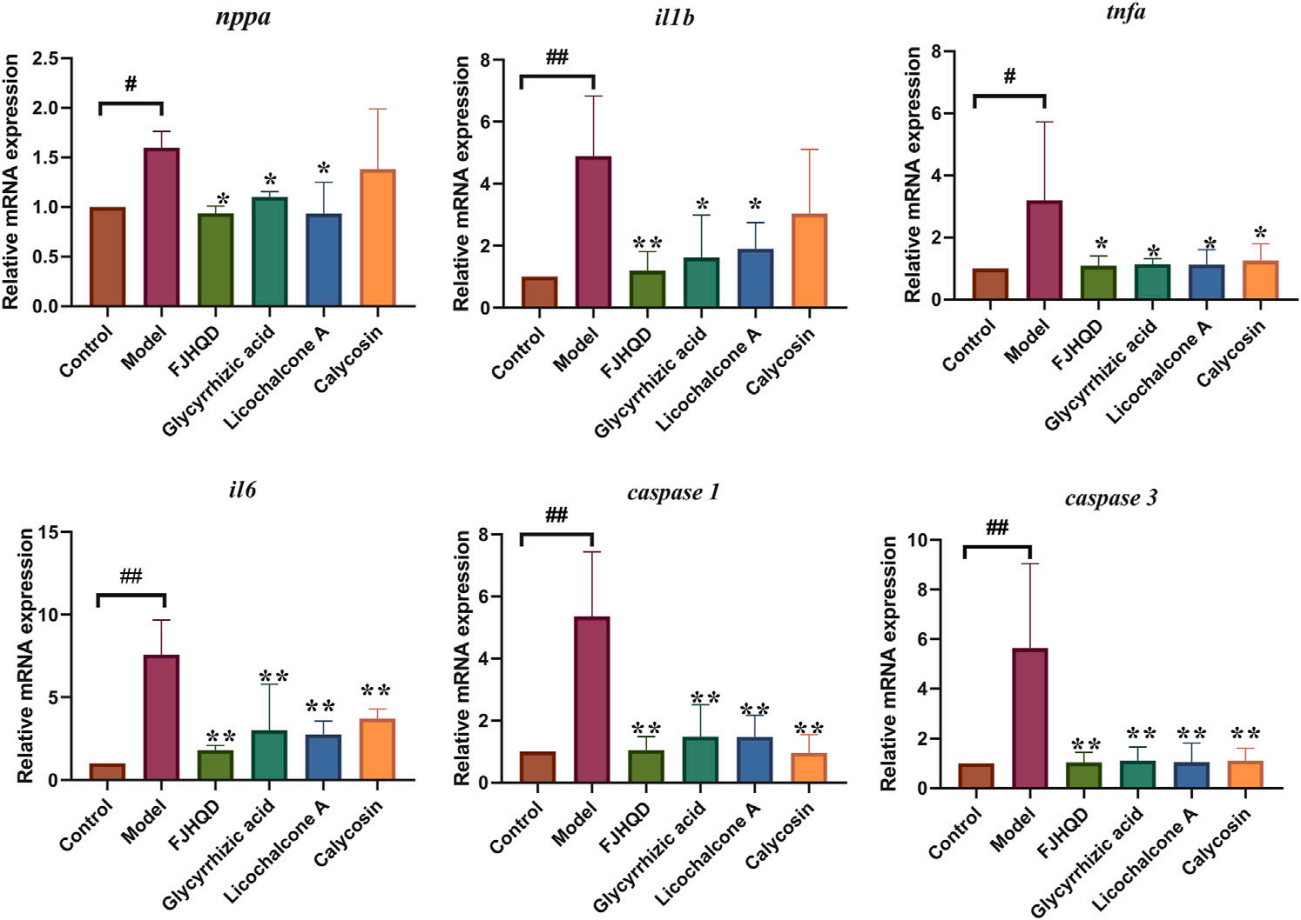
The mRNA relative expression of *nppa, tnfa, il1b, il6, caspase 1* and *caspase 3*. N = 3. ^#^ *p* < 0.05 vs*.* Control, ^##^ *p* < 0.01 vs*.* Control, * *p* < 0.05 vs*.* Model, ** *p* < 0.01 vs*.* Model.

**FIGURE 9:**
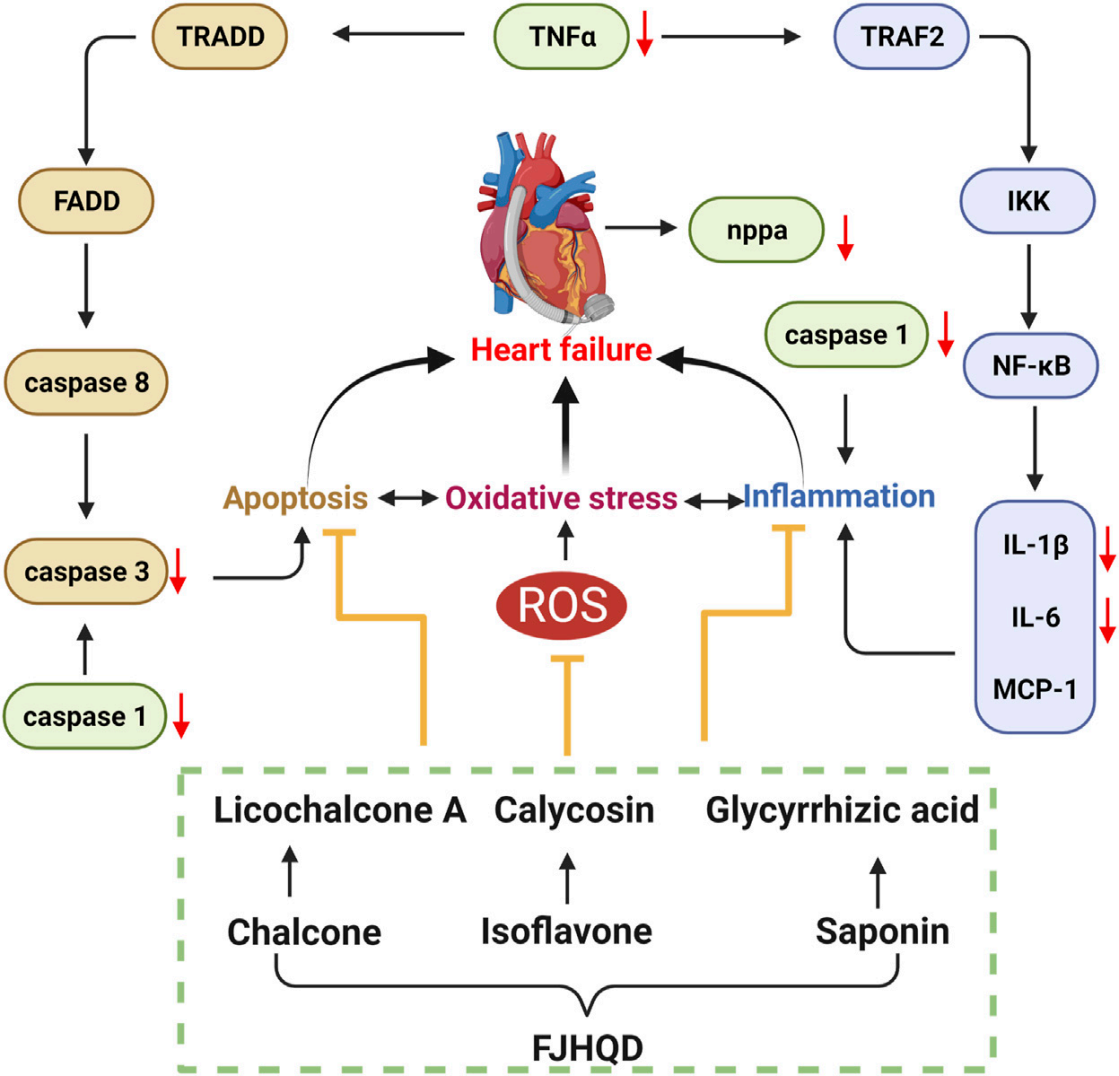
FJHQD and its active ingredients may exert anti-heart failure activity through pathways related to inflammation and apoptosis.

Excessive accumulation of ROS during the development of heart failure leads to an inflammatory response and apoptosis. In order to further investigate the mechanisms of anti-heart failure effect of FJHQD, glycyrrhizic acid, licochalcone A and calycosin, the genes of heart failure markers and pro-inflammatory factors were investigated by qPCR. As results, the transcript levels of zebrafish inflammatory factors *tnfa*, *il1b* and *il6* were all significantly increased after verapamil treatment and can be down-regulated by FJHQD, glycyrrhizic acid and licochalcone A. In addition, the expression of heart failure markers *nppa*, the caspase family of pro-inflammatory and apoptotic factors *caspase 1* and *caspase 3* also got a reduction after the treatment (Figure 8). These suggested that FJHQD and its active ingredients may exert anti-heart failure activity through pathways related to inflammation and apoptosis (Figure 9).

## Discussion

In recent years, the clinical application of FJQHD has focused on the treatment of chronic heart failure. Although FJHQD has a great therapeutic effect on heart failure, its main ingredients and mechanisms of action are still not clear. In our study, the anti-heart failure activities of FJHQD, its main botanical drugs, components and ingredients were evaluated by verapamil-induced zebrafish heart failure model.

Heart failure is a complex group of clinical syndromes caused by multiple causes of structural abnormalities and/or dysfunctions of the heart that impair the pumping function of the heart and cannot meet the metabolic needs of the body (Rossignol et al., 2019; McDonagh et al., 2021). Oxidative stress, inflammatory response and apoptosis play important roles in the development of heart failure and they interact with each other. Oxidative stress-induced ROS accumulation is thought to be a critical process in the activation of inflammatory signaling pathways. On the other hand, inflammatory responses produce more ROS, which exacerbates oxidative stress (D'Aiuto et al., 2010; Bondia-Pons et al., 2012). Our results demonstrated that FJHQD and its active ingredients can reduce ROS levels in zebrafish. Systemic inflammation has been suggested to be a common pathobiological feature of heart failure. The pro-inflammatory cytokines TNFα, IL-1β and IL-6 are all involved in the inflammatory response related to the pathogenesis of heart failure. Previous studies reported that inhibition of transcription and translation of inflammatory factors was accompanied by an improvement in cardiac function along with a decrease in plasma levels of inflammatory factors in heart failure patients (Mann, 2015). In contrast, multiple cells of the heart can secrete pro-inflammatory cytokines, leading to left ventricular dysfunction, myocardial apoptosis, and ventricular remodeling (Parrillo et al., 1989; Skudicky et al., 2000; Sliwa et al., 2002). Previous studies found that verapamil induction in zebrafish larvae elevated the levels of ROS and malondialdehyde, decreased superoxide dismutase levels, and increased the expression of *il1b*, *nf-κb*, and *tnfa* (Li et al., 2021). Our results indicated that FJHQD and the active ingredients can reduce the elevated ROS and transcript levels of inflammatory factor caused by verapamil, and we speculated that they may inhibit *il1b*, *il6*, and *tnfa* by suppressing oxidative stress in verapamil-treated zebrafish larval model.

Excess secretion of inflammatory factors can further activate caspase family proteins and subsequently initiate the apoptotic cascade response (Abbate et al., 2008). *Caspase 3* is considered to be the most important effector in the apoptosis cascade, and inhibition of *caspase 3* activation protects cardiomyocytes from ischemia or hypoxia-induced apoptosis *in vivo* and *in vitro* (Zhang et al., 2015). Caspase 1 is the core of the pro-inflammatory proteins in the caspase family, but it has also been found that *caspase 1* can induce apoptosis of cardiomyocytes by activating *caspase 3* and *caspase 9* (Merkle et al., 2007). Our results showed that verapamil caused overexpression of inflammatory factors, followed by activation of the apoptotic program in cardiomyocytes and a significant increase in the mRNA expression of *caspase 1* and *caspase 3*. However, the transcript levels of *caspase 1* and *caspase 3* were significantly reduced after pre-protection with FJHQD and its active ingredients.

Taken together, FJHQD can dose-dependently alleviate the decline of cardiac function caused by verapamil. STR, AR and GRR, as well as the components of A-STR, F-AR and F-GRR also had excellent anti-heart failure activity. Glycyrrhizic acid, licochalcone A and calycosin were the main ingredients of FJHQD that exhibit anti-heart failure activity. FJHQD and its active ingredients could significantly reduce the transcript levels of heart failure markers *nppa*, pro-inflammatory factors *il1b*, *il6*, *tnfa*, and the caspase family *caspase 1*, *caspase 3*, suggesting that FJHQD and its active ingredients may improve verapamil-induced decline in zebrafish cardiac function by regulating oxidative stress, inflammatory response and apoptosis-related pathways. Our study provides biological evidences toward the clinical efficacy in FJHQD in the management of heart failure.

## Data Availability

The original contributions presented in the study are included in the article/Supplementary Material, further inquiries can be directed to the corresponding authors.

## Abbreviation

FJHQD, Fangjihuangqi Decoction; AHFI, Anti-Heart Failure Index; STR, *Stephania Tetrandra* S.Moore [Menispermaceae; Stephaniae Tetrandrae Radix]; AR, *Astragalus mongholicus* Bunge [Fabaceae; Astragali Radix]; AMR, *Atractylodes macrocephala* Koidz. [Asteraceae; Atractylodis Macrocephalae Rhizoma]; GRR, *Glycyrrhiza uralensis* Fisch. ex DC. [Fabaceae; Glycyrrhizae Radix et Rhizoma]; A-STR, Total Alkaloids from Stephaniae Tetrandrae Radix; P-STR, Total Polysaccharide from Stephaniae Tetrandrae Radix; P-AR, Total Polysaccharide from Astragali Radix; S-AR, Total Saponins from Astragali Radix; F-AR, Total Flavonoids from Astragali Radix; P-GRR, Total Polysaccharide from Glycyrrhizae Radix et Rhizoma; F-GRR, Total Flavonoids from Glycyrrhizae Radix et Rhizoma; HR, Heart Rate; ES, End Systole; ED, End Diastole; FAC, Fractional Area Change; SV, Stroke Volume; FS, Fractional Shortening; EF, Ejection Fraction; CO, Cardiac Output; RCF, Recovery Score of Cardiac Function.
