# Supplementary material for: Screening of anti-heart failure active compounds from fangjihuangqi decoction in verapamil-induced zebrafish model by anti-heart failure index approach
Source: Front Pharmacol. 2022 Oct 7;13:999950. doi: 10.3389/fphar.2022.999950 (PMC9585168; doi:10.3389/fphar.2022.999950)
Supplement: Supplementary file 1 [file Table1.DOCX]

**Screening of Anti-Heart Failure Active Compounds from Fangjihuangqi Decoction in Verapamil-Induced Zebrafish Model by Anti-Heart Failure Index Approach**

Jun Li^1†^, Yue Zhu^1†^, Xiaoping Zhao^2*^, Lu Zhao^1,3^, Yi Wang^1,3,4,5^, Zhenzhong Yang^1, 3,4,5*^

^1^ Pharmaceutical Informatics Institute, College of Pharmaceutical Sciences, Zhejiang University, Hangzhou, China

^2^ School of Basic Medical Sciences, Zhejiang Chinese Medical University, Hangzhou, China

^3^ Innovation Institute for Artificial Intelligence in Medicine of Zhejiang University, Hangzhou, China

^4^ Jinhua Institute of Zhejiang University, Jinhua, China

^5^ Innovation Center in Zhejiang University, State Key Laboratory of Component-Based Chinese Medicine, Hangzhou, China

*** Correspondence:**Zhenzhong Yang
[yangzz@zju.edu.cn](mailto:yangzz@zju.edu.cn)

Xiaoping Zhao
[zhaoxiaoping@zcmu.edu.cn](mailto:zhaoxiaoping@zcmu.edu.cn)

^†^These authors share first authorship

**Keywords: Fangjihuangqi Decoction; Heart failure; Zebrafish; Oxidative stress; Inflammatory response; Apoptosis.**

Supplementary Material


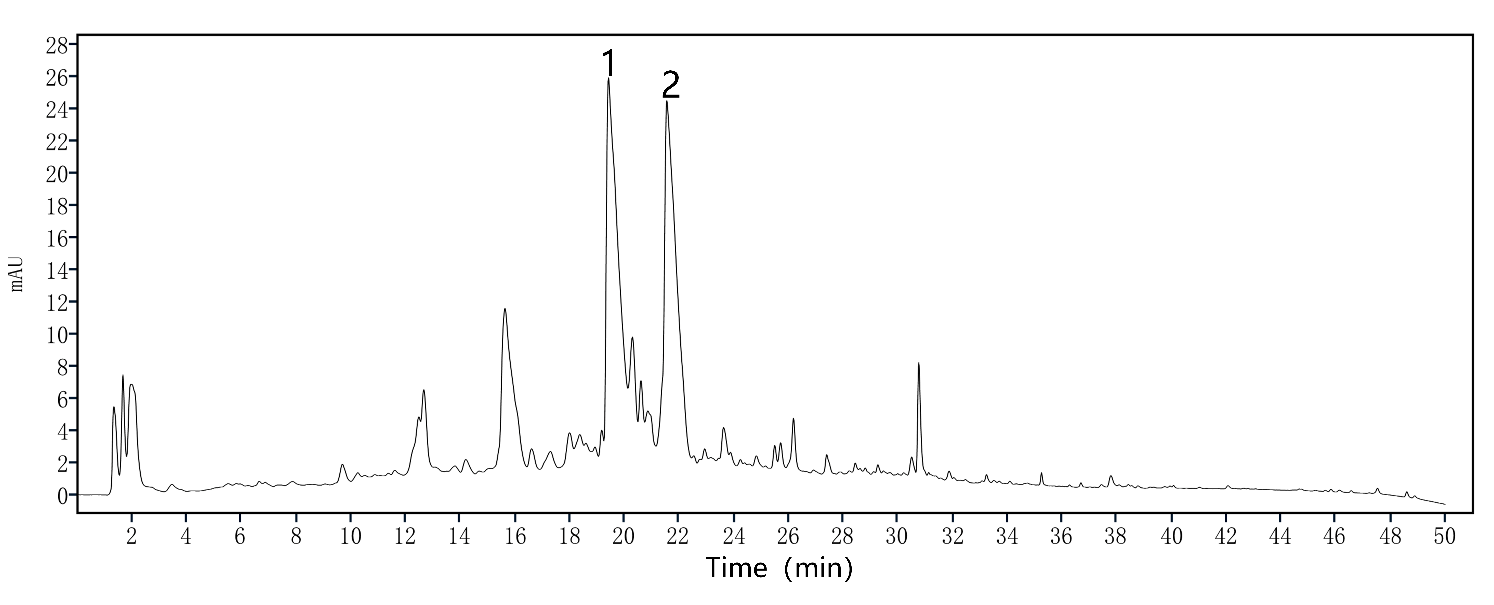


**Supplementary Figures S1**. HPLC chromatogram of A-STR. No.1 is fangchinoline, 2 is tetrandrine.


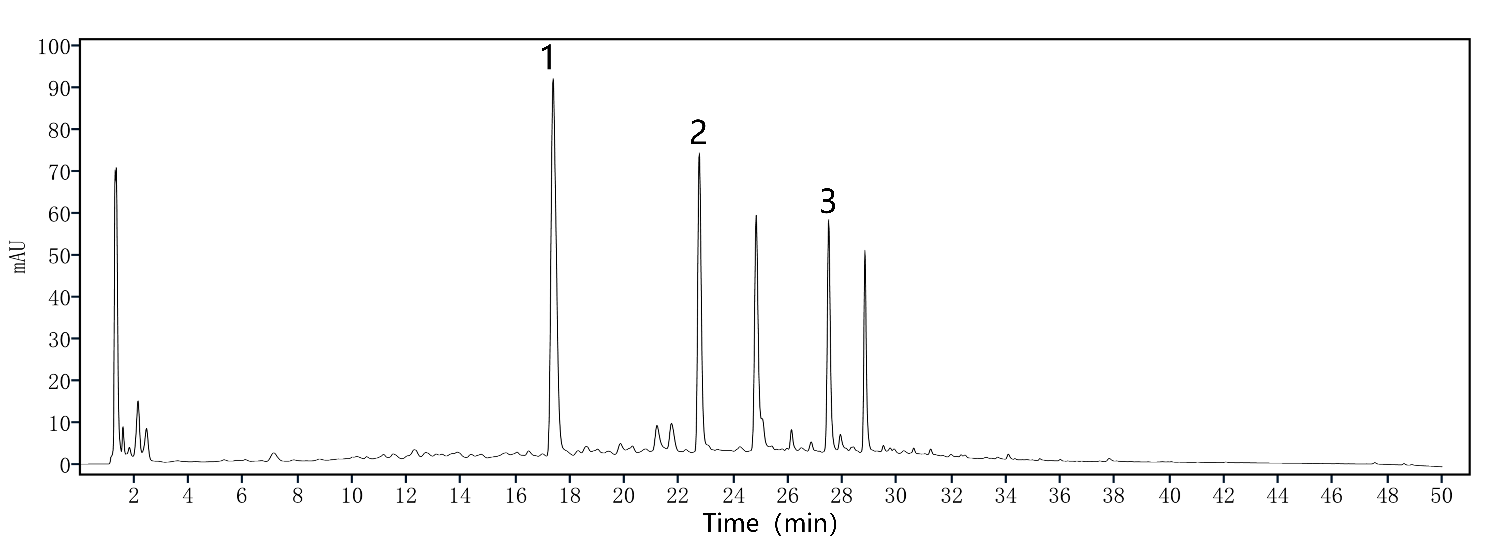


**Supplementary Figures S2**. HPLC chromatogram of F-AR. No.1 is calycosin-7-O-glucoside, 2 is ononin, 3 is calycosin.


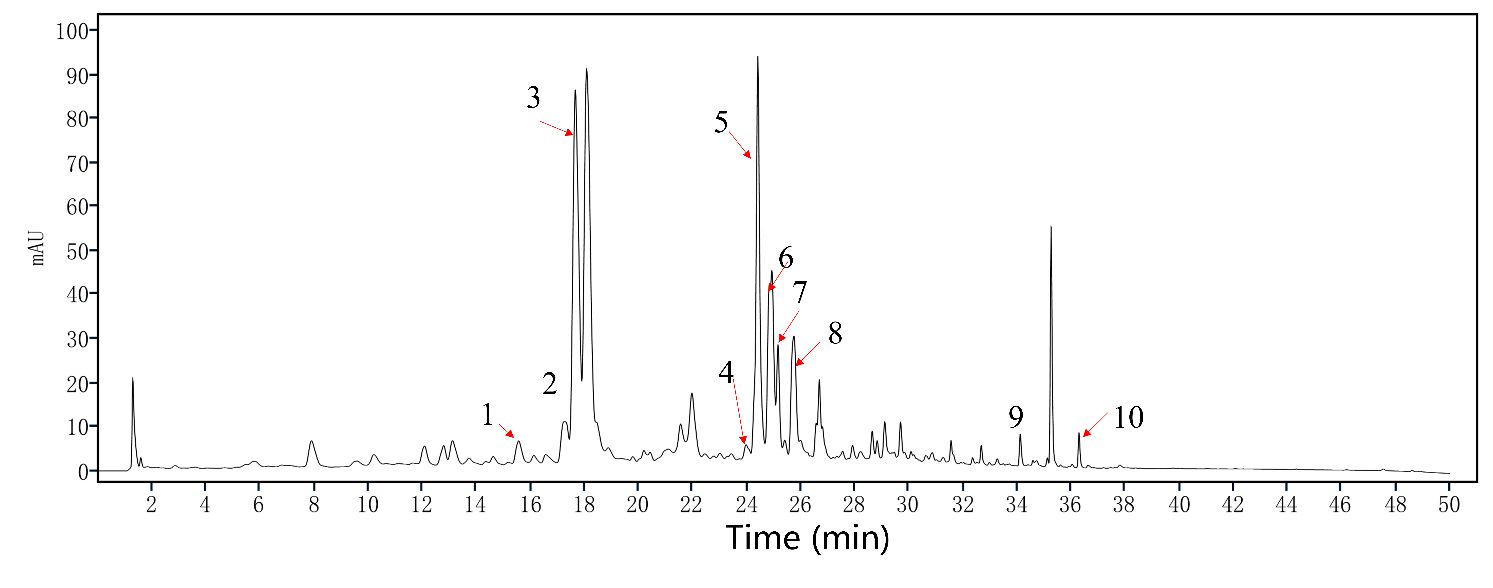


**Supplementary Figures S3**. HPLC chromatogram of F-GRR. No.1 is liquiritin, 2 is liquiritin apioside, 3 is neoliquiritin,4 is isoliquiritin apioside, 5 is isoliquiritin, 6 is neoisoliquiritin, 7 is licochalcone B, 8 is liquiritigenin, 9 is isoliquiritigenin, 10 is licochalcone A.
